# nanoDSF: *In vitro* Label-Free Method to Monitor Picornavirus Uncoating and Test Compounds Affecting Particle Stability

**DOI:** 10.3389/fmicb.2020.01442

**Published:** 2020-06-26

**Authors:** Antonio Real-Hohn, Martin Groznica, Nadine Löffler, Dieter Blaas, Heinrich Kowalski

**Affiliations:** Center for Medical Biochemistry, Max Perutz Labs, Vienna Biocenter, Medical University of Vienna, Vienna, Austria

**Keywords:** picornaviruses, rhinovirus, intrinsic tryptophan fluorescence, uncoating, pleconaril, capsid binders

## Abstract

Thermal shift assays measure the stability of macromolecules and macromolecular assemblies as a function of temperature. The Particle Stability Thermal Release Assay (PaSTRy) of picornaviruses is based on probes becoming strongly fluorescent upon binding to hydrophobic patches of the protein capsid (e.g., SYPRO Orange) or to the viral RNA genome (e.g., SYTO-82) that become exposed upon heating virus particles. PaSTRy has been exploited for studying the stability of viral mutants, viral uncoating, and the effect of capsid-stabilizing compounds. While the results were usually robust, the thermal shift assay with SYPRO Orange is sensitive to surfactants and EDTA and failed at least to correctly report the effect of excipients on an inactivated poliovirus 3 vaccine. Furthermore, interactions between the probe and capsid-binding antivirals as well as mutual competition for binding sites cannot be excluded. To overcome these caveats, we assessed differential scanning fluorimetry with a nanoDSF device as a label-free alternative. NanoDSF monitors the changes in the intrinsic tryptophan fluorescence (ITF) resulting from alterations of the 3D-structure of proteins as a function of the temperature. Using rhinovirus A2 as a model, we demonstrate that nanoDFS is well suited for recording the temperature-dependence of conformational changes associated with viral uncoating with minute amounts of sample. We compare it with orthogonal methods and correlate the increase in viral RNA exposure with PaSTRy measurements. Importantly, nanoDSF correctly identified the thermal stabilization of RV-A2 by pleconaril, a prototypic pocket-binding antiviral compound. NanoDFS is thus a label-free, high throughput-customizable, attractive alternative for the discovery of capsid-binding compounds impacting on viral stability.

## Introduction

The genus *Enterovirus* comprises many small (∼30 nm) non-enveloped icosahedral viruses belonging to the family *Picornaviridae*. It includes numerous human and animal pathogens such as rhinoviruses, poliovirus, coxsackievirus, and enterovirus 71, causing mild to life-threatening diseases like the common cold, dilated cardiomyopathy, and paralytic poliomyelitis. Their proteinaceous shell is formed from 60 copies of the four structural proteins, VP1–VP4, protecting a ∼7,100–7,500 nucleotides long (+)ssRNA genome (Jiang et al., 2014). Once bound to a cognate receptor at the host cell surface, they are taken up by endocytosis. In the endosome, the native virion expands by about 4% converting it into the subviral A particle, preparing the release of the RNA. The process is triggered by the acidic pH and/or the receptor acting as a catalyst [reviewed in Tuthill et al. (2010)]. The conformational change is preceded by the expulsion of the pocket factor, a natural lipid residing in a hydrophobic pocket in the capsid protein (Smyth and Martin, 2002) followed by the release of the internal myristoylated protein VP4 (myrVP4), and the externalization of N-terminal sequences of VP1. In the A particle, the hydrophobic pocket is collapsed, and it has channels at the 2-fold axes of icosahedral symmetry. Several medium- to high-resolution models of the expanded state of enteroviruses are available. All indicate altered contacts between the RNA and the inner surface of the capsid (Liu et al., 2011; Pickl-Herk et al., 2013; Shingler et al., 2013; Zhu et al., 2018). These changes likely facilitate the exit of the viral genome through one of the openings at the 2-fold axis, leaving behind an empty subviral B particle (Bostina et al., 2011). The exposed amphipathic N-termini of VP1, together with the extruded myrVP4, are thought to create a channel in the endocytic vesicle for the transit of the genome into the cytosol, where it initiates its replication. Native (N), A and B particles are also termed 150S, 135S, and 80S particles in the instance of rhinoviruses (Lonberg-Holm and Yin, 1973), according to their sedimentation rates in sucrose density gradients. Subviral particles and native virions also exhibit differences in electromigration in low-percentage agarose gels (Tsang et al., 2000).

Heating of enteroviral particles such as poliovirus, rhinovirus, coxsackievirus B3, echovirus 1, and enterovirus 71 has been frequently used to trigger uncoating *in vitro*, allowing for the structural characterization of the resulting subviral particles. Dependent on the temperature (37 to 60°C), time of treatment (minutes to hours), and buffer composition, this results in the preferential generation of A particles, a mixture of A and B particles, or almost pure B particles that are indistinguishable from those observed *in vivo* (Lonberg-Holm and Noble-Harvey, 1973; McGeady and Crowell, 1981; Wetz and Kucinski, 1991; Curry et al., 1996; Okun et al., 1999; Belnap et al., 2000; Shingler et al., 2013; Subirats et al., 2013; Ruokolainen et al., 2019). Adjusting the above parameters, it was also possible to isolate an intermediate conformational state of poliovirus in the process of RNA release (Bostina et al., 2011). Similar protocols have been used to trigger uncoating in other picornaviruses such as aphtho- and cardioviruses, whose capsids rather dissociate upon heating, similarly as observed *in vivo* (Bachrach, 1964; Mullapudi et al., 2016).

The above heat-induced conformational changes are also exploited for assessment of the thermal stability of picornaviruses. Due to its relative simplicity, the “Particle Stability Thermal Release” (PaSTRy) assay (Walter et al., 2012) is most often employed in the picornavirus field. In a variant of the plate-based “Differential Scanning Fluorescence” (DSF) assay (Niesen et al., 2007), a virus sample is slowly heated in a qPCR machine. Alterations of the virion structure as a function of the temperature are probed with fluorescent dyes; SYPRO Orange or SYPRO Red, whose fluorescence is quenched when free in solution, bind to hydrophobic patches normally buried inside the capsid but becoming accessible by conformational changes (often loosely termed unfolding) during temperature ramping, leading to a ∼500-fold increase in their emission intensity. A melting temperature T_m_, determined at the transition midpoint of the plotted data, serves as a virus-stability measure. Exposure of the viral genome can be detected with nucleic acid intercalating dyes such as Midori Green (Hankaniemi et al., 2019), SYBR Green II, SYTO-9 (Walter et al., 2012), and SYTO-82 (Wald et al., 2019). Here, the mid-point transition temperature T_R_ (or ET_50_) is interpreted as a measure for the viral RNA becoming accessible for the probe as a consequence of capsid expansion (allowing the dye to enter the particle and interact with the genome) or as 50% of the RNA released from the particle (Walter et al., 2012; Schotte et al., 2014). However, these two cannot be differentiated without additional experiments.

The PaSTRy assay has been used to assess the impact of pH, ions, antibodies, recombinant receptors, and mutations on the thermal stability of picornaviral capsids (Walter et al., 2012; Rayaprolu et al., 2013; Shakeel et al., 2015; Zhu et al., 2018). PaSTRy was also employed for quantifying the effect of novel capsid-binding anti-viral drug candidates [e.g., Wald et al. (2019)]. However, SYPRO dyes can give rise to a high fluorescence already at low temperatures as observed with certain virus samples (Qi et al., 2014), which is also the case for nucleic acid intercalating dyes when the native capsids are already permeable (Tuthill et al., 2009). Furthermore, drug candidates and certain excipients may potentially interact with the fluorescent probes or compete for binding, complicating interpretation of the results; EDTA associates with SYPRO Orange at alkaline pH leading to artifacts (Kroeger et al., 2017) and SYPRO dyes are incompatible with surfactants (Ablinger et al., 2013).

In order to avoid the above shortcomings, the intrinsic fluorescence of tryptophan (TRP) in biomolecules can be utilized instead. Intrinsic tryptophan fluorescence (ITF) detects changes in the protein microenvironment based on the solvatochromic characteristics of the indole ring. Upon excitation, at 280 nm, the TRP fluorescence emission maximum is at around 330 nm when located in an apolar environment. In a polar environment, the TRP emission intensity usually decreases due to static and dynamic quenching by solvent molecules, and the peak emission is red-shifted to about 350 nm (Duy and Fitter, 2006; Zahid et al., 2012). This is typically the case when TRP residues hidden in the hydrophobic core of a protein become exposed to water upon its denaturation/unfolding. Less drastic allosteric changes may result in red- or blue-shifted emission of the involved TRP residues dependent on the resultant local environment. The temperature required for the unfolding of 50% of a protein, or of a given domain, can be precisely determined from the extent of red-shift (or occasionally blue-shift) over the applied temperature gradient in a label-free manner.

Nanoscale differential scanning fluorimetry (nanoDSF) allows for high-precision detection of dual-UV intrinsic fluorescence changes resulting from thermal unfolding of proteins in low volume capillaries (Alexander et al., 2014). By applying nanoDSF measurements on RV-A2, one of the many common cold-causing rhinoviruses (for a recent review see Basnet et al., 2019) as an example, we demonstrate that ITF can provide a detailed picture of the heat-induced *in vitro* uncoating of an enterovirus in real-time. In addition, using pleconaril as a capsid binding antiviral, we qualify nanoDSF as a tool with the potential to high-throughput applications as an alternative to PaSTRy, for the label-free thermostability analysis of picornaviruses.

## Materials and Methods

### Virus and Reagents

All experiments were carried out with RV-A2, initially acquired from ATCC and propagated, purified, and polished via monolithic chromatography as described previously (Allmaier et al., 2018). The presence of natural empty particles was evaluated by separating 10 μg of purified RV-A2 on a 15% SDS-polyacrylamide gel; no significant amount of VP0, indicative of natural empties, was detected (Supplementary Figure S2b). SYTO-82 was purchased from Thermo Fisher, pleconaril from Merck.

### Nanoscale Differential Scanning Fluorometry (nanoDSF)

Real-time simultaneous monitoring of the ITF at 330 nm and 350 nm during the conversion of native RV-A2 into subviral A and B particles was carried out in a Prometheus NT.48 instrument from NanoTemper Technologies with an excitation wavelength of 280 nm (Magnusson et al., 2019). Capillaries were filled with 10 μl of a suspension of RV-A2 (1 mg/ml in PBS), placed into the sample holder and the temperature was increased from 25 to 95°C at a ramp rate of 1°C/min, with one fluorescence measurement per 0.044°C. The ratio of the recorded emission intensities (Em_350nm_/Em_330nm_), which represents the change in TRP fluorescence intensity as well as the shift of the emission maximum to higher wavelengths (“red-shift”) or lower wavelengths (“blue-shift”) was plotted as a function of the temperature. Since the changes relevant to viral uncoating occurred between 40 and 65°C, only this temperature range is depicted in the corresponding figures. The fluorescence intensity ratio and its first derivative were calculated with the manufacturer’s software (PR.ThermControl, version 2.1.2). For assaying virus particle stabilization, purified RV-A2 was mixed with a stock solution of pleconaril in DMSO to give final concentrations of 0.5 mg/ml, 1 mM, and 2%, respectively. In control1, the pleconaril was omitted (=solvent control); in control2, the pleconaril solution was replaced with water. Three independent measurements were carried out for each condition, and their mean is depicted.

### Particle Stability Thermal Release Assay (PaSTRy)

The PaSTRy was performed as in Walter et al. (2012) with minor adaptations. Briefly, the temperature at which the viral RNA became accessible for SYTO-82 was determined in a Bio-Rad CFX Connect Real-Time PCR instrument. Purified RV-A2 at 0.5 mg/ml was supplemented with SYTO-82 to a concentration of 5 μM in a final volume of 70 μl PBS. Each measurement was carried out in triplicate with 20 μl aliquots. Samples were transferred into wells of a thin-walled PCR plate, and the temperature was ramped from 25 to 95°C, 1.5°C/min (Wald et al., 2019). At each 0.5°C step, the temperature was maintained for 5 s, plus an additional 15 s for reading. The fluorescence emission mean from three independent measurements at 560 nm (excitation at 541 nm) was plotted as a function of the temperature. Again, raw fluorescence and its normalized first derivative are displayed only for the temperature range 40–65°C.

### Negative Stain Electron Microscopy

Virus samples were diluted to 0.5 mg/ml in PBS and heated at 1°C/min to 52 or 55°C, cooled to 4°C, and 8 μl were applied to glow discharged carbon-coated grids and left for 10 min. The grids were washed with 2% sodium phosphotungstic acid (PTA), pH 7.6, followed by 10 min incubation with PTA and blotting with filter paper. The stained samples were imaged in a FEI Morgagni electron microscope (80 kV, 110 k magnification). Identification of (sub)viral particles was as in Kumar and Blaas (2013); native virions appear as impermeable bright spheres. A particles are permeable for the stain, making them appear as bright rings with a grainy interior. Empty B particles present as bright circles encasing a uniformly dark core stemming from the negative stain accumulating in the void (Figure 2D - white arrows). In total, ten distinct fields from two independent treatments were evaluated (∼1000 particles per treatment), and the mean and standard deviation calculated. Two-way ANOVA with multiple correlation analysis reveals that the difference from 52 to 55°C is a statistically significant change in the population of all the subviral particles (*p* < 0.0001).

## Results

### Number and Positions of Tryptophan Residues in RV-A2

**FIGURE 1:**
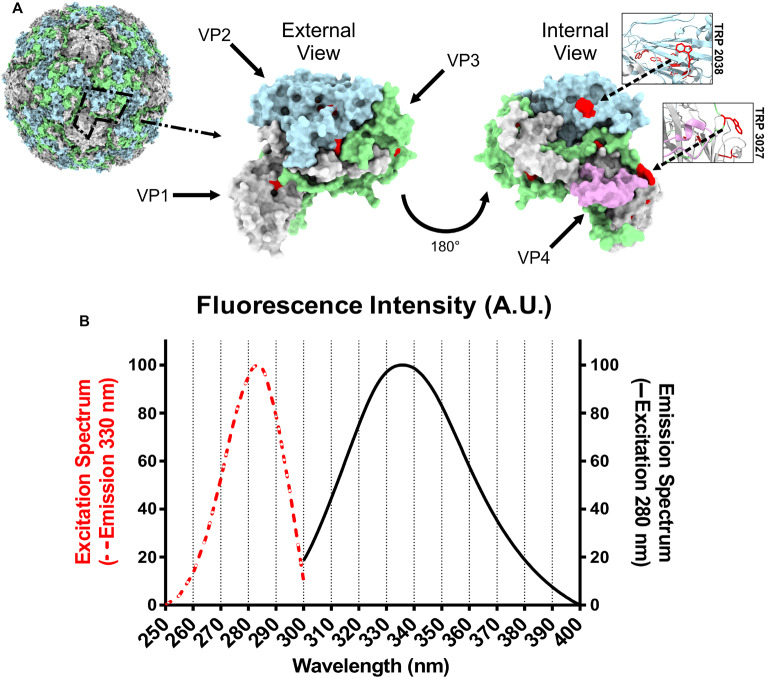
Positions of RV-A2 tryptophan residues within a biological protomer and fluorescence spectra of RV-A2. **(A)** Left: 3D-structure surface-view (pdb:1FPN) with one protomer highlighted with a dashed black line. The solvent accessible surface is rendered by using a rolling sphere of 1.4 Å radius in Chimera. Middle and right: Surface of an isolated protomer viewed from within and from without the capsid. VP1, VP2, VP3, and VP4 are colored gray, blue, green, and rose, respectively. Tryptophan residues are depicted in red; the locations of TRP2038 and TRP3027 are additionally highlighted in the boxed ribbon diagrams representing the respective local tertiary structure. **(B)** Fluorescence spectra of RV-A2 in PBS. Normalized excitation (250–300 nm) and emission (300 – 400 nm) spectra were recorded in 1 nm steps. The measurement was repeated eight times, and the mean is shown. A.U., arbitrary units.

RV-A2 is built from 12 pentamers with each one made of 5 protomers. Each protomer consists of the viral proteins VP1, VP2, VP3, and VP4. The 3D model of native RV-A2 (PDB 1FPN) shows 15 TRPs per protomer, resulting in 900 TRPs per virion. VP4, the smallest capsid protein (68 aa), ejected during the conversion of the native virion into the A particle, and VPg (21 aa), covalently linked to the 5′-end of the encapsidated genomic RNA, lack TRP. Visual inspection of an individual protomer (Figure 1A), demonstrates that thirteen TRPs (red) are largely buried and inaccessible to water. Of the remaining two TRPs, TRP 2038 (the first digit denotes the viral protein, here VP2) is at the inner surface of the capsid, stacking against one or two bases of the viral RNA (Verdaguer et al., 2000). As seen in other enterovirus 3D-structures, this interaction is strictly conserved (Hadfield et al., 1997). The second one, TRP 2027, is located at the interface with other protomers at the 5-fold axis (see also the Amino Acid Information in http://viperdb.scripps.edu).

Intrinsic protein fluorescence is due to the aromatic amino acids, mainly tryptophan (TRP), as phenylalanine (PHE) has a very low quantum yield, and emission by tyrosine (TYR) is often quenched. TRP fluorescence is furthermore most strongly influenced by the polarity of its local microenvironment and is thus the best sensor for detecting changes in its exposure to solvent. The excitation spectrum of RV-A2 suggests that only TRP residues contribute to the emission at 330 nm (dashed red line in Figure 1B); the impact of other fluorescent amino acids, specifically the 34 tyrosines (λ_em_ 303 nm) per protomer, to the overall emission spectrum was insignificant, even though TRP and TYR have an excitation maximum close to 280 nm. The emission maximum at 335 nm (solid black line in Figure 1B) indicated a rather apolar environment for most TRPs in the native virus. This is consistent with their majority being located at hydrophobic cores of the 8-stranded beta-barrels formed by each of the structural proteins VP1, VP2, and VP3 (Figure 1A).

### Viral Uncoating Examined by nanoDSF and PaSTRy

**FIGURE 2:**
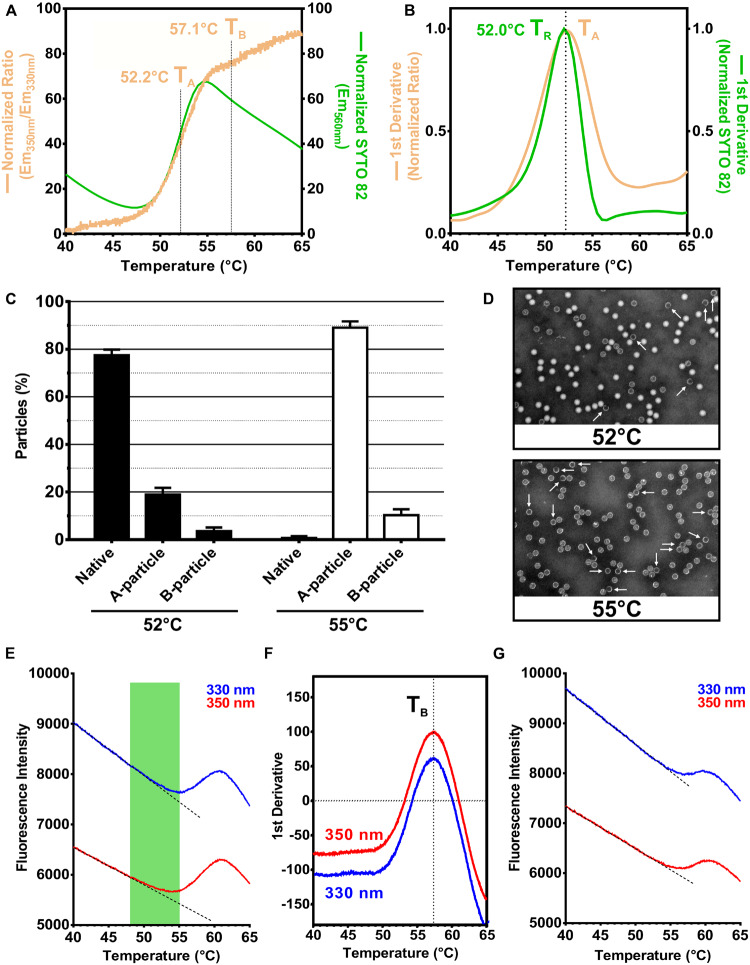
RV-A2 nanoDSF and PaSTRy analysis and correlation with negative stain ultrastructure analysis. **(A)** RV-A2 in PBS (1 mg/ml) was analyzed by nanoDSF by heating from 25 to 95°C with a ramp rate of 1°C/min. Samples were excited at 280 nm, and the intensity of the TRP emission at 330 and 350 nm was recorded for every ∼0.02°C temperature increase. In the PaSTRy assay, the virus preparation (0.5 mg/ml) was heated in a real-time PCR machine from 25 to 95°C at a 1.5°C/min ramp rate. Samples were excited at 541 nm, and the emission intensity was determined at 560 nm at each 0.5°C temperature increase. The obtained data were plotted against the temperature, with the left *Y*-axis indicating the TRP fluorescence emission ratio as the intensity at 350 nm/intensity at 330 nm (curve is shown in orange) and the right *Y*-axis the normalized SYTO-82 emission intensity at 560 nm (curve is shown in green). **(B)** First derivatives calculated from the experimental data shown in panel **(A)**. All curves (in panels **A,B**) were normalized to their individual minimum and maximum values using GraphPad Prism 6.01 for better comparison and represent the mean of 3 independent thermal scans; only the temperature range relevant for the heat-triggered virus uncoating is shown. T_A_ is the temperature where 50% of N into A particle conversion takes place, T_B_ corresponds to 50% conversion of (full) A into empty B particles. **(C)** RV-A2 in PBS was subject to the same thermal gradient as in the nanoDSF analysis, using a real-time PCR machine instead. The heating was terminated at 52 and 55°C, and the samples were quickly placed on ice followed by particle imaging via negative stain transmission electron microscopy. A, B and remaining N particles were counted and identified based on their differential dye penetrability. The percentage of (sub)viral particles in each class is presented as a bar graph for each temperature, normalized to the total number of native particles before heating. In total, ten distinct fields were evaluated, and the mean and standard deviation calculated. **(D)** Representative micrographs at a magnification of 110 kx (B particles are indicated with white arrows). **(E)** Graphs of the 350 and 330 nm TRP emission of heated RV-A2 as used for the ITF ratio calculation in panel **(A)**. Dotted straight lines represent the pretransition baseline obtained by data fitting in each instance. The temperature window encompassing the TRP red-shift as a consequence of the N into A particle conversion is highlighted in green. For both curves (blue, red) a drastic sigmoid increase in TRP intensity is visible thereafter (from about 55 to 60°C), which is not evident in a distinct red-shift and thus the Em_350nm_/Em_330nm_ ratio. Notably, the onset of the sigmoid part in both instances (E_330_ and E_350_) practically coincides with the peak of the SYTO-82 signal (55°C) in panel **(A)**. **(F)** Graphs of the first derivative of the data in panel **(E)**. T_B_, which is identical for both maxima, corresponds to the temperature where 50% of A particles transformed into B particles. The N into A conversion identified by the increase in red-shift is not visible as a discrete maximum in this plot. **(E)** Purified RV-A2 was heated at 56°C for 10 min resulting in the formation of mostly B particles, which were then analyzed by nanoDSF identically as outlined in panel **(A)** for the native virus. In the obtained raw 350 and 330 nm emission curves, a slight sigmoidal transition is evident from 57 to 60°C. As expected, the maximum at 60°C coincides with the maximum for the A into B particle conversion. Dotted straight lines represent the respective pretransition baselines obtained by data fitting.

Next, we analyzed the change in ITF of RV-A2 with increasing temperature in a nanoDSF device. An alteration in the microenvironment of TRP residues is typically revealed by a change in the ratio of the emission fluorescence intensity at 350 and 330 nm; tyrosines minimally contribute to the signal. The Em_350nm_/Em_330nm_ ratio is also a direct measure of the relative abundance of different molecular species present in the sample (Kotov et al., 2019; Krakowiak et al., 2019). The ratio of fluorescence at 350 and 330 nm modestly increased until ∼47°C (Figure 2A, orange curve). This slight red-shift might be attributed to augmented breathing of the protein shell, which starts between 25 and 37°C dependent on the type of the virus (Li et al., 1994; Katpally et al., 2009). This capsid breathing allows RNA binding dyes to enter the native virion through transiently opening pores. However, even at 37°C, substantial labeling required several hours of incubation (Kremser et al., 2004). Entry of SYTO-82 into native virus can thus be disregarded at the much shorter time scale of the nanoDSF assay (∼1 h). This is also indicated by the low normalized SYTO-82 fluorescence recorded in the PaSTRy experiment carried out in parallel (Figure 2A, green curve). At temperatures beyond 47°C, the ITF ratio (Figure 2A, orange curve) as well as the SYTO 82 signal (Figure 2A, green curve) suddenly increased. Such sigmoidal curves are usually observed for a 2-state unfolding mechanism (Consalvi et al., 2000). However, at temperatures well below the denaturation of the many individual proteins making up the virus, the signal must rather be due to a concerted and prominent conformational change occurring within this temperature window. Decrease of the SYTO 82 signal beyond 55°C is probably due to dissociation of the dye from the nucleic acid. The inflection points of the curves are remarkably similar despite the two methods measuring ITF and RNA exposure, respectively (Figure 2B).

In order to get quantitative insight into the population distribution of the different (sub)viral particles present within the temperature window of the cooperative transition shown in Figure 2A, we heated native RV-A2 in a real-time PCR machine at the identical ramp rate of 1°C/min as used in nanoDSF. Samples were collected at 52°C (close to the midpoint of the transition) and 55°C (at the peak of the sigmoid part of the graph). N, A, and B particles present at these temperatures were then revealed by negative stain transmission electron microscopy (TEM). As shown in Figures 2C,D, the slow heating of RV-A2 to 52°C resulted in the appearance of ∼20% A and 3% empty B particles, while the majority (77%) remained native. These proportions dramatically changed at 55°C, where most of the heated virus had converted into subviral, mostly A particles (90%) and just ∼10% B particles, with less than 1% of “surviving” native virions. From these data, we conclude that the sigmoid transition observed in the plot of the TRP fluorescence ratio versus the temperature corresponds predominantly to the conformational conversion of native virus to A particles. This was supported by the PaSTRy experiment (Figure 2A, green curve), which showed an almost superimposable rise in the normalized SYTO-82 emission intensity. The A particles, due to their porosity, allow unrestricted binding of intercalating dyes to their genomic RNA (Liu et al., 2018; Ruokolainen et al., 2019), which evidently occurs at the same pace as new A particles are formed from the native virus. Based on these results we define the midpoint temperature of the Em_350nm_/Em_330nm_ ratio (its value at the inflection point), and precisely determined at the maximum of its first derivative (Figure 2B, orange curve), as the temperature T_A_, where 50% of native RV-A2 converted into subviral A particles. As expected, this temperature was practically identical to the temperature T_R_, where 50% of viral RNA genomes became accessible to SYTO-82. It was determined from the maximum of the first derivative of the Em_560nm_ versus temperature graph (52.0°C, green curve, vs. 52.2°C, orange curve; Figure 2B).

The abrupt increase in the Em_350nm_/Em_330nm_ ratio likely indicates that the swelling of the shell during the N to A particle conversion moved one or more TRP residues to a more polar (aqueous) microenvironment. Inspection of the high-resolution 3D structure of native RV-A2 (Verdaguer et al., 2000) and the derived A particles (Pickl-Herk et al., 2013) suggests TRP3027 as a key candidate for this bathochromic effect. It is located at interprotomeric sites in the native virion. In the A particle, TRP3027 becomes more exposed to water that penetrates through the pores opening in the expanded capsid, while the other TRP residues appear much less affected (Supplementary Figures S1b vs. S1d).

We did not detect a distinct second transition in the plot of the Em_350nm_/Em_330nm_ as a function of temperature for the conversion of A into empty B particles. This observation is in line with the small differences in the structure of their shells: a comparison of the X-ray models shows only a minor displacement of the VP2 N-terminal region (residues 46–55) and the VP3 F–G loop (residues 149–162), although the first 61 N-terminal amino acid residues of VP1 (devoid of tryptophan) might be retracted and disordered in the B particle (Garriga et al., 2012; Pickl-Herk et al., 2013). Hence, all TRP residues in the two structures including TRP3027 must be in a very similar microenvironment (Supplementary Figures S1d,f), explaining the lack of a distinct red-shift for the conversion of the A into the B particle. Nevertheless, attempting to capture even subtle conformational changes, we plotted the individual fluorescence intensities recorded at Em_330nm_ and Em_350nm_ against the temperature (Figure 2E). The graph obtained at 350 nm (red curve) showed a lesser intensity decay than the one recorded at 330 nm (blue curve). This is in line with the TRP red-shift seen for the temperature range of the cooperative conversion of N into A particles (region highlighted in green). Strikingly, upon further raising the temperature, the intensity at both emission wavelengths drastically increased, starting at 55°C and peaking at 60°C. The inflection point of this sigmoidal transition, which lacks a red-shift and is thus invisible in the Em_350nm_/Em_330nm_ ratio (Figure 2A), was at the same temperature (T_B_ = 57.1°C), for both wavelengths, as determined from the maxima of the respective first derivatives (Figure 2F).

The temperature of onset of the above transition corresponded to the peak in the SYTO-82 signal (Figure 2A, green curve); we thus surmised that the subsequent sigmoid part might relate to the transformation of full (RNA containing) A into empty B particles. Therefore, we performed a nanoDSF analysis using RV-A2 heated for 10 min to 56°C, yielding mostly B particles as previously reported (Harutyunyan et al., 2013). The individual Em_330nm_ and Em_350nm_ curves for this sample (Figure 2G) showed a considerably lesser rise from 55 to 60°C as compared to the corresponding traces in Figure 2E. We thus believe that it is related to some residual A particles present in this preparation. However, the peak of the S-shaped part of the curves was at the same temperature (i.e., 60°C, Figures 2E,G), suggesting the formation of a uniform population of empty particles.

That the conversion of A into empty B particles resulted in a sharp intensity increase in the raw fluorescence data was, at first sight, unexpected. Revisiting the structures of the A and empty B particles with respect to their tryptophans revealed one distinct difference for TRP2038, which is situated in the N-terminal segment of VP2 facing the interior of the capsid. Because of the corresponding high density of the averaged map it is likely that each of the 60 VP2 copies in the native RV-A2 virion is engaged in a stacking interaction with the viral genome (Verdaguer et al., 2000) and Supplementary Figure S1b; stacking denoted with an asterisk. Stacking of TRP with nucleobases has previously been shown to efficiently quench its emission as a result of charge transfer from the indole ring to the purine or pyrimidine rings (Helene et al., 1971; Brun et al., 1975). Though slightly displaced, the TRP2038 base stacking interaction is maintained in the cryo-EM and X-ray maps of subviral A particles produced by incubation of RV-A2 in acidic buffer (Supplementary Figure S1d; stacking denoted with an asterisk). Furthermore, it has been shown that TRP stacking is maintained until at least 50°C (Toulme and Helene, 1977). Taken together, it is thus tempting to speculate that the fluorescence of TRP2038 is quenched in both N and A particles even at temperatures above 37°C. Upon ejection of the viral RNA from the A particle this stacking is lost and would, consequently, recover the TRP2038 fluorescence, conceivably explaining the observed intensity increase without a marked red-shift, due to its otherwise unchanged polar microenvironment.

Since the SYTO-82 fluorescence remained significantly above background, the expelled viral RNA must still be partially double-stranded as previously observed for similar temperatures in melting experiments with UV absorbance recording of picornaviral RNA *in situ* and *in vitro* (Bachrach, 1964; Frisby et al., 1977). The progressive loss in the SYTO-82 signal intensity on further temperature increase is hence most likely related to dissociation of the dye from the released genomic RNA upon the gradual denaturation of the remaining secondary structures in concert with the continuous decrease due to thermal quenching (Zaboikin et al., 2018).

The conformational switch of various members of the *Enterovirus* genus from N to A and/or B particles is an irreversible process and strictly under kinetic control (Tsang et al., 2000; Harutyunyan et al., 2013). At neutral pH and in the presence of stabilizing ions the conversions occur between 50 and 60°C. Furthermore, for proper analysis of irreversible multistate unfolding of proteins in thermal shift assays, a scan rate of 1°C/min is frequently chosen (Strucksberg et al., 2007; Senisterra et al., 2012). In order to assess a possible influence of the heating speed on the onset of the conformational transformations, RV-A2 aliquots were ramped within 1 min from room temperature to the temperatures given in Supplementary Figure S2a followed by immediate cooling. The generated subviral particles were then assessed by agarose gel electrophoresis. As shown in Supplementary Figure S2a, this fast heating resulted in the transition from N to A particles around 50°C and from A to B particles at a slightly higher temperature.

### RV-A2 Capsid Stabilization by Pleconaril Evaluated by nanoDSF

**FIGURE 3:**
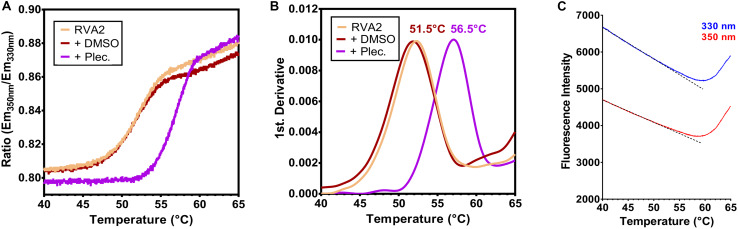
nanoDSF demonstrates the thermostabilization of RV-A2 by pleconaril. Purified RV-A2 was diluted in PBS (1 mg/ml) ± 1 mM pleconaril (Plec.) and analyzed by nanoDSF. The heating rate was 1°C/min and tryptophan fluorescence was recorded in steps of ∼0.02°C. **(A)** Ratio Em_350nm_/Em_330nm_ plotted against the temperature. The steeper sigmoidal curve in the presence of pleconaril relates to the faster transition of native (N) to A particles at a higher temperature after the drug has been thermally expelled from the pocket. Orange trace, untreated virus; brown trace, DMSO treated virus (solvent control “+DMSO”); purple trace, pleconaril treated virus (“+Plec”). **(B)** The first derivative of the curves in panel **(A)**. The temperature where 50% of the virus has converted into A particles is indicated, which is shifted by ∼5°C to the right in the presence of the capsid-stabilizing compound. The curves represent the mean of three independent measurements. **(C)** Raw 350 and 330 nm emission curves of purified RV-A2 treated with pleconaril used for ITF Ratio calculation in panel **(A)**. Dotted straight lines represent the pretransition baseline obtained by data fitting. The overall profile is similar to Figure 2C, though the onset of A into B particle conversion (the left minimum of the Em_330nm_ and Em_350nm_ curves) is shifted by about 5°C to higher temperatures.

We then measured the effect of pleconaril on the thermostability of RV-A2. This compound inserts with high affinity into the hydrophobic pocket within the viral capsid protein VP1 of many enteroviruses, including rhinovirus species A and B. The pocket is beneath the floor of the canyon, a cleft surrounding the 5-fold axis (Rossmann et al., 1985; Smith et al., 1986). Such capsid-binders stabilize the viral capsid, which in turn, blocks uncoating (Lewis et al., 1998) and/or docking to the cellular receptor (Dewindt et al., 1994). Compared to DMSO as solvent control, which had no significant impact on the stability of RV-A2 (Figure 3A), pleconaril shifted the temperature T_A_, where 50% of virus particles convert into A particles, from 51.5 to 56.5°C as determined from the maximum of the first derivative of the ITF ratio of the fluorescence signals (Figure 3B). The native state of RV-A2 is maintained by pleconaril up to higher temperatures as clearly illustrated by a constant Em_350nm_/Em_330nm_ ratio. Upon expulsion of the pleconaril native virions become converted into subviral A particles (Tsang et al., 2000). The steeper sigmoid slope observed for the pleconaril-treated RV-A2, as compared to curves for the controls (untreated and DMSO-treated virus) indicates that the transition is faster when shifted to a higher temperature. The subsequent conversion of A into empty B particles is again only evident in the raw emission signals (compare Figure 3C with Figure 2E). In accordance, the T_A_ values in the absence/presence of pleconaril were practically identical to the respective T_R_ values previously determined for RV-A2 by PaSTRy with SYTO-82, measuring RNA exposure (Figure 2H in Wald et al., 2019).

## Discussion

We examined nanoDSF as a complementary technique for the label-free monitoring of heat-induced virus uncoating and assessed its potential suitability for the screening of capsid-binding viral inhibitors. NanoDSF revealed, in real-time, the conversion of native RV-A2 virions into full subviral A particles and their subsequent transformation (at ∼5°C higher temperature) into empty B particles. This is particularly interesting as A and B particles have an almost identical subviral protein shell. Detection of both transitions was achieved by applying two different options for ITF data analysis. Importantly, the sequential conversion was confirmed by negative stain TEM of the particle population collected after exposure to different temperatures in the first sigmoidal transition interval in combination with nanoDSF of enriched B particles. As the same sequence of events is also observed for *in vivo* uncoating, our ITF analysis lends further support to the usefulness of controlled heating of enterovirus as appropriate *in vitro* model for mimicking this process. To our surprise, we found only four previous reports using ITF for the recording of conformational changes in gradually heated enteroviruses, though employing different instruments and all focusing on poliovirus (a member of the C-type enteroviruses) (Grimmel et al., 1983; Chen et al., 1997; Qi et al., 2014; Qi et al., 2018). The outcome of these studies differs from ours by the absence of a recognizable transition associated with B particle formation, the end product of enterovirus uncoating. Furthermore, in no instance was ultrastructure analysis used for proper quantification of uncoating intermediates arising during the temperature ramping.

Intrinsic tryptophan fluorescence has been used in the past to study discrete changes in the capsid of the parvovirus minute virus of mice subjected to a similar thermal gradient. The authors found two clearly separated sigmoidal cooperative transitions, one for externalization of the N-terminus of VP1 (akin to enteroviruses) at lower temperature and the second being associated with dissociation of the virion and release of its linear, ssDNA genome at a considerably higher temperature. Both transitions were observed in the same fluorescence intensity curve (Reguera et al., 2005). By contrast, determination of the change in red-shift (expressed as Em_350nm_/Em_330nm_ ratio), as well as the change in the raw intrinsic fluorescence intensity, was required to reveal the sigmoid N to A and A to B particle transitions for RV-A2 by nanoDSF. Furthermore, these cooperative transformations occurred in an almost overlapping fashion even at slow temperature ramping and without marked capsid dissociation as verified by TEM. The exit of viral RNA hence most likely occurred via its transient unfolding promoted at a higher temperature in order to pass through a small pore in the subviral shell as previously described for isothermally heated (56°C) RV-A2 and PV1 (Levy et al., 2010; Harutyunyan et al., 2013; Shingler et al., 2013).

RV-A2 possesses 900 TRPs in its icosahedral capsid. The static intrinsic fluorescence emission spectrum reflects the average molecular environment of the 15 tryptophan residues in each protomer. Though reducing the complexity, contributions from individual tryptophans are normally challenging to deconvolute. However, an inspection of the high-resolution 3D structure of native RV-A2 (Verdaguer et al., 2000), and the derived A (Pickl-Herk et al., 2013) and B (Garriga et al., 2012) particles provided important clues for the nanoDSF data evaluation. Of all indole rings, the one of TRP3027 sitting at the edge of a protomer becomes most exposed to the solvent on the expansion of the capsid during the conversion of N (Supplementary Figures S1a,b) into A particles (Supplementary Figures S1c,d), likely explaining the accompanying sharp red-shift. The distinct sigmoidal rise in the raw fluorescence plots with little red-shift [due to their very similar shell interiors (Supplementary Figures S1d,f)] characterizing the A to B particle transition might conceivably result from dequenching of TRP2038 when its stacking interaction with the viral RNA is lost upon exit of the latter from the capsid. An increase of emission intensity without any red-shift due to removal of quenching was recently described for TRP residues involved in conformational changes of monomeric transthyretin (Jazaj et al., 2019). However, while the TRP2038–RNA stacking interaction is highly conserved in other enteroviruses (Hadfield et al., 1997), a similar sudden increase of the intrinsic fluorescence intensity is not visible in the respective graphs of four thermostability analyses previously done by other researchers with PV1 or PV3 over a similar temperature range as employed in our nanoDSF (Grimmel et al., 1983; Chen et al., 1997; Qi et al., 2014, 2018). This finding is quite puzzling, as the PV RNA also exited the capsid in these studies, which should consequently result in an analogous destacking and release of the equivalent TRP2038 from fluorescence quenching by the nucleobase. A possible reason might be a perhaps suboptimal stacking interaction of TRP2038 with the PV RNA, resulting in an inefficient quenching in the full particles. In fact, a previous study on a phage protein – RNA enhancer interaction demonstrated that the fluorescence quenching of an intercalating TRP depended critically on the type of the stacking base and the internal RNA architecture (Su et al., 1997). Alternatively, the increase in TRP fluorescence intensity during the A to B particle transition of RV-A2 might originate from the removal of quenching by a different event not obvious in the corresponding 3D models. NanoDSF of a broader range of enteroviruses, including *in vitro* mutagenesis of TRP2038, should help to clarify this point, although, in a preliminary study, such mutants displayed severely reduced viability (Hadfield et al., 1997).

We note that a recent cryo-EM analysis indicated that A particle formation by exposure of the acid-labile enterovirus EV-D68 to a mildly acidic pH seems to proceed via an equally expanded and fenestrated E1 particle intermediate, which has not externalized the VP1 N-termini from the capsid interior nor expelled VP4 (Liu et al., 2018). This novel intermediate remains to be identified for RV-A2; if (transiently) formed during temperature ramping, it is evidently not apparent as a separate sigmoid transition in the TRP red-shift data nor the emission intensity plot. Also, due to a similar porosity, it cannot be distinguished from “classical” A particles in negative stain TEM. Cryo-EM analysis of PV1 heated to 56°C to promote B (80S) particle formation by the Hogle lab, and our fluorescence correlation spectroscopy study on equally heated RV-A2 furthermore indicated that RNA release is gradual, likely proceeding via a series of expanded 80S-like intermediates characterized by different degrees of RNA exit (Levy et al., 2010; Harutyunyan et al., 2013). However, in the nanoDSF fluorescence intensity data, the conversion of full A into empty B particles appears as a simple cooperative two-state transition. This does not automatically imply that such intermediate structures are non-existent, but rather that these states are not observable by the applied experimental techniques (ITF, TEM) as is discussed in a broader context in El-Baba et al. (2016).

For each sigmoid rise in the nanoDSF data (corresponding to N into A and A into B particle conversion), due to the high density of the acquired data points, we could very precisely determine mid-point temperatures, T_A_ and T_B_ from the slope of the respective first derivatives (Figures 2A,B,F). The normalized curves for the Em_350nm_/Em_330nm_ ratio and the SYTO 82 emission intensity independently determined by PaSTRy were furthermore practically superimposable, yielding nearly identical mid-point transition temperatures for RNA exposure (T_R_) and A particle formation (T_A_). The SYTO 82 signal reached a peak at a temperature where the A into B particle conversion, and consequently expulsion of the genomic RNA, has just commenced according to the second transition only found in the raw TRP fluorescence curves and our TEM analysis. Hence, at least under our conditions, T_R_ indicates 50% N to A particle transition and not 50% RNA release, as occasionally claimed from the 50% fluorescence intensity increase of nucleic acid binding dyes in similar thermal shift assays using other enteroviruses [e.g., in Schotte et al. (2014)], despite lacking considerable experimental support.

Taken together, compared to PaSTRy, intrinsic tryptophan fluorescence (ITF) measurements can provide more in-depth insight into the temperature-dependent conformational transitions typical of enteroviruses, going beyond the rather ill-characterized capsid unfolding event detected by SYPRO dyes. The high sensitivity of nanoDSF for monitoring of ITF enables analysis at low sample concentrations and thereby substantially reduces the risk of unspecific heat-induced aggregation. This is particularly important for those picornaviruses, which give rise to hydrophobic A particle uncoating intermediates as is the case for polio- and rhinoviruses due to the exposure of amphipathic N-terminal extensions of VP1 (Lonberg-Holm et al., 1976; Fricks and Hogle, 1990). We hence believe that nanoDSF will substantially facilitate future investigations addressing the role of capsid-RNA interactions in coordinated uncoating of the viral genome as suggested from cryo-EM analysis (Pickl-Herk et al., 2013), similarly as described in Reguera et al. (2005).

Because of the low sample consumption, we regard nanoDSF also suitable as a high throughput screening tool for rapid identification and evaluation of capsid-binding antiviral compounds as exemplified here with pleconaril. Treatment of RV-A2 with this compound resulted in a highly reproducible shift of the mid-point transition temperature T_A_ to a 5°C higher value, very close to the one reported in a recently published study obtained with SYTO 82 in a PaSTRy analysis (Wald et al., 2019). The effect of pleconaril could be easily attributed to thermal stabilization of the N particle (Figure 3), in concert with its published mechanism of action [reviewed in Florea et al. (2003)]. In a broader context, nanoDSF can also serve as a potent alternative for rapid screening of the impact of pH, ions, and excipients on the uncoating of virus particles and/or their physical stability. Indeed, ITF has proved to be superior to PaSTRy with SYPRO Orange for establishing the thermal stability profile of an inactivated PV3 vaccine in a recent preformulation study (Qi et al., 2014). The advantage of nanoDSF compared with SYPRO Orange was also demonstrated in a very recent thermostability analysis for discrimination of adeno-associated virus serotypes, which are small, icosahedral ssDNA viruses belonging to the parvovirus family (Rieser et al., 2020). This is also the only other study we are aware of having used this technique for native virus analysis. Though not tested here, thermal stability analysis with nanoDSF is not restricted to enteroviruses but also an option with other picornaviruses. ITF has been previously used for examining the effect of ions on the thermal stability of hepatitis A virus (Volkin et al., 1997) and in another report for assessing the impact of pressure on the integrity of the capsid of foot-and mouth-disease virus (Oliveira et al., 1999). Finally, another advantage of nanoDSF over PaSTRy is the avoidance of potential interactions of test compounds with the fluorescent dyes used in the latter method and/or competition for the same binding sites; it further does not prohibit adding surfactants to the assay. On the downside, quenching of tryptophans or overlapping excitation/emission by added substances cannot be excluded, which is usually not a severe problem in PaSTRy. Ideally, usage of both methods in parallel should be considered when planning to carry out a robust high-throughput antiviral compound screen.

## Data Availability Statement

All datasets presented in this study are included in the article/supplementary material.

## Author Contributions

AR-H, DB, and HK designed the study and wrote the manuscript. AR-H, MG, and NL conducted experiments and analyzed and interpreted the data. All authors contributed to the article and approved the submitted version.

## Conflict of Interest

The authors declare that the research was conducted in the absence of any commercial or financial relationships that could be construed as a potential conflict of interest.

## References

[B1] Ablinger E. Leitgeb S. Zimmer A. (2013). Differential scanning fluorescence approach using a fluorescent molecular rotor to detect thermostability of proteins in surfactant-containing formulations. *Int. J. Pharm.* 441 255–260. 10.1016/j.ijpharm.2012.11.035 23200956

[B2] Alexander C. G. Wanner R. Johnson C. M. Breitsprecher D. Winter G. Duhr S. (2014). Novel microscale approaches for easy, rapid determination of protein stability in academic and commercial settings. *Biochim. Biophys. Acta* 1844 2241–2250. 10.1016/j.bbapap.2014.09.016 25262836 PMC4332417

[B3] Allmaier G. Blaas D. Bliem C. Dechat T. Fedosyuk S. Gosler I. (2018). Monolithic anion-exchange chromatography yields rhinovirus of high purity. *J. Virol. Methods* 251 15–21. 10.1016/j.jviromet.2017.09.027 28966037 PMC5694342

[B4] Bachrach H. L. (1964). Foot-and-mouth disease virus: structure and mechanism of degradation as deduced from absorbance-temperature relationships. *J. Mol. Biol.* 8 348–358. 10.1016/s0022-2836(64)80198-4 14168688

[B5] Basnet S. Palmenberg A. C. Gern J. E. (2019). Rhinoviruses and their receptors. *Chest* 155 1018–1025. 10.1016/j.chest.2018.12.012 30659817 PMC6533451

[B6] Belnap D. M. Filman D. J. Trus B. L. Cheng N. Booy F. P. Conway J. F. (2000). Molecular tectonic model of virus structural transitions: the putative cell entry states of poliovirus. *J. Virol.* 74 1342–1354. 10.1128/jvi.74.3.1342-1354.2000 10627545 PMC111469

[B7] Bostina M. Levy H. Filman D. J. Hogle J. M. (2011). Poliovirus RNA is released from the capsid near a twofold symmetry axis. *J. Virol.* 85 776–783. 10.1128/JVI.00531-10 20980499 PMC3020038

[B8] Brun F. Toulme J. J. Helene C. (1975). Interactions of aromatic residues of proteins with nucleic acids. Fluorescence studies of the binding of oligopeptides containing tryptophan and tyrosine residues to polynucleotides. *Biochemistry* 14 558–563. 10.1021/bi00674a015 234245

[B9] Chen C. H. Wu R. Roth L. G. Guillot S. Crainic R. (1997). Elucidating mechanisms of thermostabilization of poliovirus by D2O and MgCl2. *Arch. Biochem. Biophys.* 342 108–116. 10.1006/abbi.1997.0111 9185619

[B10] Consalvi V. Chiaraluce R. Giangiacomo L. Scandurra R. Christova P. Karshikoff A. (2000). Thermal unfolding and conformational stability of the recombinant domain II of glutamate dehydrogenase from the hyperthermophile *Thermotoga maritima*. *Protein Eng.* 13 501–507. 10.1093/protein/13.7.501 10906345

[B11] Curry S. Chow M. Hogle J. M. (1996). The poliovirus 135S particle is infectious. *J. Virol.* 70 7125–7131. 10.1128/jvi.70.10.7125-7131.1996 8794359 PMC190765

[B12] Dewindt B. van Eemeren K. Andries K. (1994). Antiviral capsid-binding compounds can inhibit the adsorption of minor receptor rhinoviruses. *Antiviral Res.* 25 67–72. 10.1016/0166-3542(94)90094-9 7811059

[B13] Duy C. Fitter J. (2006). How aggregation and conformational scrambling of unfolded states govern fluorescence emission spectra. *Biophys. J.* 90 3704–3711. 10.1529/biophysj.105.078980 16500981 PMC1440751

[B14] El-Baba T. J. Kim D. Rogers D. B. Khan F. A. Hales D. A. Russell D. H. (2016). Long-lived intermediates in a cooperative two-state folding transition. *J. Phys. Chem. B* 120 12040–12046. 10.1021/acs.jpcb.6b08932 27933943

[B15] Florea N. R. Maglio D. Nicolau D. P. (2003). Pleconaril, a novel antipicornaviral agent. *Pharmacotherapy* 23 339–348. 10.1592/phco.23.3.339.32099 12627933 PMC7168037

[B16] Fricks C. E. Hogle J. M. (1990). Cell-induced conformational change in poliovirus: externalization of the amino terminus of VP1 is responsible for liposome binding. *J. Virol.* 64 1934–1945. 10.1128/jvi.64.5.1934-1945.1990 2157861 PMC249347

[B17] Frisby D. Cotter R. I. Richards B. (1977). Structural studies of encephalomyocarditis virus RNA both in situ and in free solution. *J. Gen. Virol.* 37 311–322. 10.1099/0022-1317-37-2-311 200710

[B18] Garriga D. Pickl-Herk A. Luque D. Wruss J. Caston J. R. Blaas D. (2012). Insights into minor group rhinovirus uncoating: the X-ray structure of the HRV2 empty capsid. *PLoS Pathog.* 8:e1002473. 10.1371/journal.ppat.1002473 22241997 PMC3252380

[B19] Grimmel M. Zibirre R. Koch G. (1983). Fluorescence spectrophotometric study of structural alterations in the capsid of poliovirus. *Arch. Virol.* 78 191–201. 10.1007/bf01311314 6318693

[B20] Hadfield A. T. Lee W. Zhao R. Oliveira M. A. Minor I. Rueckert R. R. (1997). The refined structure of human rhinovirus 16 at 2.15 A resolution: implications for the viral life cycle. *Structure* 5 427–441. 10.1016/s0969-2126(97)00199-8 9083115

[B21] Hankaniemi M. M. Stone V. M. Sioofy-Khojine A. B. Heinimaki S. Marjomaki V. Hyoty H. (2019). A comparative study of the effect of UV and formalin inactivation on the stability and immunogenicity of a Coxsackievirus B1 vaccine. *Vaccine* 37 5962–5971. 10.1016/j.vaccine.2019.08.037 31471148

[B22] Harutyunyan S. Kumar M. Sedivy A. Subirats X. Kowalski H. Kohler G. (2013). Viral uncoating is directional: exit of the genomic RNA in a common cold virus starts with the poly-(A) tail at the 3′-end. *PLoS Pathog.* 9:e1003270. 10.1371/journal.ppat.1003270 23592991 PMC3617019

[B23] Helene C. Dimicoli J. L. Brun F. (1971). Binding of tryptamine and 5-hydroxytryptamine (serotonin) to nucleic acids. Fluorescence and proton magnetic resonance studies. *Biochemistry* 10 3802–3809. 10.1021/bi00796a025 5107012

[B24] Jazaj D. Ghadami S. A. Bemporad F. Chiti F. (2019). Probing conformational changes of monomeric transthyretin with second derivative fluorescence. *Sci. Rep.* 9:10988. 10.1038/s41598-019-47230-4 31358790 PMC6662758

[B25] Jiang P. Liu Y. Ma H. C. Paul A. V. Wimmer E. (2014). Picornavirus morphogenesis. *Microbiol. Mol. Biol. Rev.* 78 418–437. 10.1128/MMBR.00012-14 25184560 PMC4187686

[B26] Katpally U. Fu T. M. Freed D. C. Casimiro D. R. Smith T. J. (2009). Antibodies to the buried N terminus of rhinovirus VP4 exhibit cross-serotypic neutralization. *J. Virol.* 83 7040–7048. 10.1128/JVI.00557-09 19403680 PMC2704786

[B27] Kotov V. Bartels K. Veith K. Josts I. Subhramanyam U. K. T. Gunther C. (2019). High-throughput stability screening for detergent-solubilized membrane proteins. *Sci. Rep.* 9:10379. 10.1038/s41598-019-46686-8 31316088 PMC6637136

[B28] Krakowiak J. Krajewska M. Wawer J. (2019). Monitoring of lysozyme thermal denaturation by volumetric measurements and nanoDSF technique in the presence of N-butylurea. *J. Biol. Phys.* 45 161–172. 10.1007/s10867-019-09521-9 30903354 PMC6548760

[B29] Kremser L. Okun V. M. Nicodemou A. Blaas D. Kenndler E. (2004). Binding of fluorescent dye to genomic RNA inside intact human rhinovirus after viral capsid penetration investigated by capillary electrophoresis. *Anal. Chem.* 76 882–887. 10.1021/ac034898x 14961716

[B30] Kroeger T. Frieg B. Zhang T. Hansen F. K. Marmann A. Proksch P. (2017). EDTA aggregates induce SYPRO orange-based fluorescence in thermal shift assay. *PLoS One* 12:e0177024. 10.1371/journal.pone.0177024 28472107 PMC5417642

[B31] Kumar M. Blaas D. (2013). Human rhinovirus subviral a particle binds to lipid membranes over a twofold axis of icosahedral symmetry. *J. Virol.* 87 11309–11312. 10.1128/JVI.02055-13 23946453 PMC3807301

[B32] Levy H. C. Bostina M. Filman D. J. Hogle J. M. (2010). Catching a virus in the act of RNA release: a novel poliovirus uncoating intermediate characterized by cryo-electron microscopy. *J. Virol.* 84 4426–4441. 10.1128/JVI.02393-09 20181687 PMC2863768

[B33] Lewis J. K. Bothner B. Smith T. J. Siuzdak G. (1998). Antiviral agent blocks breathing of the common cold virus. *Proc. Natl. Acad. Sci. U.S.A.* 95 6774–6778. 10.1073/pnas.95.12.6774 9618488 PMC22631

[B34] Li Q. Yafal A. G. Lee Y. M. Hogle J. Chow M. (1994). Poliovirus neutralization by antibodies to internal epitopes of VP4 and VP1 results from reversible exposure of these sequences at physiological temperature. *J. Virol.* 68 3965–3970. 10.1128/jvi.68.6.3965-3970.1994 7514682 PMC236902

[B35] Liu F. Liu Q. Cai Y. Leng Q. Huang Z. (2011). Construction and characterization of an infectious clone of coxsackievirus A16. *Virol. J.* 8:534. 10.1186/1743-422X-8-534 22165961 PMC3283524

[B36] Liu Y. Sheng J. van Vliet A. L. W. Buda G. van Kuppeveld F. J. M. Rossmann M. G. (2018). Molecular basis for the acid-initiated uncoating of human enterovirus D68. *Proc. Natl. Acad. Sci. U.S.A.* 115 E12209–E12217. 10.1073/pnas.1803347115 30530701 PMC6310856

[B37] Lonberg-Holm K. Gosser L. B. Shimshick E. J. (1976). Interaction of liposomes with subviral particles of poliovirus type 2 and rhinovirus type 2. *J. Virol.* 19 746–749. 10.1128/jvi.19.2.746-749.1976 183023 PMC354908

[B38] Lonberg-Holm K. Noble-Harvey J. (1973). Comparison of in vitro and cell-mediated alteration of a human Rhinovirus and its inhibition by sodium dodecyl sulfate. *J. Virol.* 12 819–826. 10.1128/jvi.12.4.819-826.1973 4359954 PMC356699

[B39] Lonberg-Holm K. Yin F. H. (1973). Antigenic determinants of infective and inactivated human rhinovirus type 2. *J. Virol.* 12 114–123. 10.1128/jvi.12.1.114-123.1973 4126195 PMC355237

[B40] Magnusson A. O. Szekrenyi A. Joosten H. J. Finnigan J. Charnock S. Fessner W. D. (2019). nanoDSF as screening tool for enzyme libraries and biotechnology development. *FEBS J.* 286 184–204. 10.1111/febs.14696 30414312 PMC7379660

[B41] McGeady M. L. Crowell R. L. (1981). Proteolytic cleavage of VP1 in ‘A’particles of coxsackievirus B3 does not appear to mediate virus uncoating by HeLa cells. *J. Gen. Virol.* 55 439–450. 10.1099/0022-1317-55-2-439 6270273

[B42] Mullapudi E. Novacek J. Palkova L. Kulich P. Lindberg A. M. van Kuppeveld F. J. (2016). Structure and genome release mechanism of the human cardiovirus saffold virus 3. *J. Virol.* 90 7628–7639. 10.1128/JVI.00746-16 27279624 PMC4988150

[B43] Niesen F. H. Berglund H. Vedadi M. (2007). The use of differential scanning fluorimetry to detect ligand interactions that promote protein stability. *Nat. Protoc.* 2 2212–2221. 10.1038/nprot.2007.321 17853878

[B44] Okun V. M. Blaas D. Kenndler E. (1999). Separation and biospecific identification of subviral particles of human rhinovirus serotype 2 by capillary zone electrophoresis. *Anal. Chem.* 71 4480–4485. 10.1021/ac990503r 10546529

[B45] Oliveira A. C. Ishimaru D. Goncalves R. B. Smith T. J. Mason P. Sa-Carvalho D. (1999). Low temperature and pressure stability of picornaviruses: implications for virus uncoating. *Biophys. J.* 76 1270–1279. 10.1016/S0006-3495(99)77290-5 10049311 PMC1300107

[B46] Pickl-Herk A. Luque D. Vives-Adrian L. Querol-Audi J. Garriga D. Trus B. L. (2013). Uncoating of common cold virus is preceded by RNA switching as determined by X-ray and cryo-EM analyses of the subviral A-particle. *Proc. Natl. Acad. Sci. U.S.A.* 110 20063–20068. 10.1073/pnas.1312128110 24277846 PMC3864292

[B47] Qi W. Orgel S. Francon A. Randolph T. W. Carpenter J. F. (2018). Urea improves stability of inactivated polio vaccine serotype 3 during lyophilization and storage in dried formulations. *J. Pharm. Sci.* 107 2070–2078. 10.1016/j.xphs.2018.04.019 29709487

[B48] Qi W. Zeng Y. Orgel S. Francon A. Kim J. H. Randolph T. W. (2014). Preformulation study of highly purified inactivated polio vaccine, serotype 3. *J. Pharm. Sci.* 103 140–151. 10.1002/jps.23801 24282078

[B49] Rayaprolu V. Kruse S. Kant R. Venkatakrishnan B. Movahed N. Brooke D. (2013). Comparative analysis of adeno-associated virus capsid stability and dynamics. *J. Virol.* 87 13150–13160. 10.1128/JVI.01415-13 24067976 PMC3838259

[B50] Reguera J. Grueso E. Carreira A. Sanchez-Martinez C. Almendral J. M. Mateu M. G. (2005). Functional relevance of amino acid residues involved in interactions with ordered nucleic acid in a spherical virus. *J. Biol. Chem.* 280 17969–17977. 10.1074/jbc.M500867200 15728575

[B51] Rieser R. Penaud-Budloo M. Bouzelha M. Rossi A. Menzen T. Biel M. (2020). Intrinsic differential scanning fluorimetry for fast and easy identification of adeno-associated virus serotypes. *J. Pharm. Sci.* 109 854–862. 10.1016/j.xphs.2019.10.031 31639391

[B52] Rossmann M. G. Arnold E. Erickson J. W. Frankenberger E. A. Griffith J. P. Hecht H. J. (1985). Structure of a human common cold virus and functional relationship to other picornaviruses. *Nature* 317 145–153. 10.1038/317145a0 2993920

[B53] Ruokolainen V. Domanska A. Laajala M. Pelliccia M. Butcher S. J. Marjomaki V. (2019). Extracellular albumin and endosomal ions prime enterovirus particles for uncoating that can be prevented by fatty acid saturation. *J. Virol.* 93:e00599-19. 10.1128/JVI.00599-19 31189702 PMC6694817

[B54] Schotte L. Strauss M. Thys B. Halewyck H. Filman D. J. Bostina M. (2014). Mechanism of action and capsid-stabilizing properties of VHHs with an in vitro antipolioviral activity. *J. Virol.* 88 4403–4413. 10.1128/JVI.03402-13 24501405 PMC3993733

[B55] Senisterra G. Chau I. Vedadi M. (2012). Thermal denaturation assays in chemical biology. *Assay Drug Dev. Technol.* 10 128–136. 10.1089/adt.2011.0390 22066913

[B56] Shakeel S. Westerhuis B. M. Ora A. Koen G. Bakker A. Q. Claassen Y. (2015). Structural basis of human parechovirus neutralization by human monoclonal antibodies. *J. Virol.* 89 9571–9580. 10.1128/JVI.01429-15 26157123 PMC4542383

[B57] Shingler K. L. Yoder J. L. Carnegie M. S. Ashley R. E. Makhov A. M. Conway J. F. (2013). The enterovirus 71 A-particle forms a gateway to allow genome release: a cryoEM study of picornavirus uncoating. *PLoS Pathog.* 9:e1003240. 10.1371/journal.ppat.1003240 23555253 PMC3605244

[B58] Smith T. J. Kremer M. J. Luo M. Vriend G. Arnold E. Kamer G. (1986). The site of attachment in human rhinovirus 14 for antiviral agents that inhibit uncoating. *Science* 233 1286–1293. 10.1126/science.3018924 3018924

[B59] Smyth M. S. Martin J. H. (2002). Picornavirus uncoating. *Mol. Pathol.* 55 214–219. 10.1136/mp.55.4.214 12147709 PMC1187181

[B60] Strucksberg K. H. Rosenkranz T. Fitter J. (2007). Reversible and irreversible unfolding of multi-domain proteins. *Biochim. Biophys. Acta* 1774 1591–1603. 10.1016/j.bbapap.2007.09.005 17964867

[B61] Su L. Radek J. T. Labeots L. A. Hallenga K. Hermanto P. Chen H. (1997). An RNA enhancer in a phage transcriptional antitermination complex functions as a structural switch. *Genes Dev.* 11 2214–2226. 10.1101/gad.11.17.2214 9303537 PMC275392

[B62] Subirats X. Weiss V. U. Gosler I. Puls C. Limbeck A. Allmaier G. (2013). Characterization of rhinovirus subviral A particles via capillary electrophoresis, electron microscopy and gas phase electrophoretic mobility molecular analysis: part II. *Electrophoresis* 34 1600–1609. 10.1002/elps.201200686 23483563

[B63] Toulme J. J. Helene C. (1977). Specific recognition of single-stranded nucleic acids. Interaction of tryptophan-containing peptides with native, denatured, and ultraviolet-irradiated DNA. *J. Biol. Chem.* 252 244–249. 556724

[B64] Tsang S. K. Danthi P. Chow M. Hogle J. M. (2000). Stabilization of poliovirus by capsid-binding antiviral drugs is due to entropic effects. *J. Mol. Biol.* 296 335–340. 10.1006/jmbi.1999.3483 10669591

[B65] Tuthill T. J. Groppelli E. Hogle J. M. Rowlands D. J. (2010). Picornaviruses. *Curr. Top. Microbiol. Immunol.* 343 43–89. 10.1007/82_2010_37 20397067 PMC3018333

[B66] Tuthill T. J. Harlos K. Walter T. S. Knowles N. J. Groppelli E. Rowlands D. J. (2009). Equine rhinitis A virus and its low pH empty particle: clues towards an aphthovirus entry mechanism? *PLoS Pathog.* 5:e1000620. 10.1371/journal.ppat.1000620 19816570 PMC2752993

[B67] Verdaguer N. Blaas D. Fita I. (2000). Structure of human rhinovirus serotype 2 (HRV2). *J. Mol. Biol.* 300 1179–1194. 10.1006/jmbi.2000.3943 10903863

[B68] Volkin D. B. Burke C. J. Marfia K. E. Oswald C. B. Wolanski B. Middaugh C. R. (1997). Size and conformational stability of the hepatitis A virus used to prepare VAQTA, a highly purified inactivated vaccine. *J. Pharm. Sci.* 86 666–673. 10.1021/js960475h 9188048

[B69] Wald J. Pasin M. Richter M. Walther C. Mathai N. Kirchmair J. (2019). Cryo-EM structure of pleconaril-resistant rhinovirus-B5 complexed to the antiviral OBR-5-340 reveals unexpected binding site. *Proc. Natl. Acad. Sci. U.S.A.* 116 19109–19115. 10.1073/pnas.1904732116 31462495 PMC6754595

[B70] Walter T. S. Ren J. Tuthill T. J. Rowlands D. J. Stuart D. I. Fry E. E. (2012). A plate-based high-throughput assay for virus stability and vaccine formulation. *J. Virol. Methods* 185 166–170. 10.1016/j.jviromet.2012.06.014 22744000 PMC3470038

[B71] Wetz K. Kucinski T. (1991). Influence of different ionic and pH environments on structural alterations of poliovirus and their possible relation to virus uncoating. *J. Gen. Virol.* 72 2541–2544. 10.1099/0022-1317-72-10-2541 1655960

[B72] Zaboikin M. Freter C. Srinivasakumar N. (2018). Gaussian decomposition of high-resolution melt curve derivatives for measuring genome-editing efficiency. *PLoS One* 13:e0190192. 10.1371/journal.pone.0190192 29300734 PMC5754072

[B73] Zahid N. I. Abou-Zied O. K. Hashim R. Heidelberg T. (2012). Fluorescence probing of the temperature-induced phase transition in a glycolipid self-assembly: hexagonal ↔ micellar and cubic ↔ lamellar. *Langmuir* 28 4989–4995. 10.1021/la3001976 22364590

[B74] Zhu L. Sun Y. Fan J. Zhu B. Cao L. Gao Q. (2018). Structures of Coxsackievirus A10 unveil the molecular mechanisms of receptor binding and viral uncoating. *Nat. Commun.* 9:4985. 10.1038/s41467-018-07531-0 30478256 PMC6255764

