# Supplementary material for: nanoDSF: In vitro Label-Free Method to Monitor Picornavirus Uncoating and Test Compounds Affecting Particle Stability
Source: Front Microbiol. 2020 Jun 26;11:1442. doi: 10.3389/fmicb.2020.01442 (PMC7333345; doi:10.3389/fmicb.2020.01442)
Supplement: Supplementary file 1 [file Data_Sheet_1.docx]

**Supplemental Methods**

**Electrophoretic analysis of the subviral particles obtained by fast heating**. A horizontal 0.5% agarose gel in TBE buffer (89 mM Tris Base, 89 mM Boric Acid, and 2 mM EDTA, pH 8) was prepared in a BioRad mini gel chamber, and the chamber filled with TBE buffer. Purified RV-A2 was diluted in PBS to a final concentration of 1 mg/ml. Aliquots of 10 µl were then heated for 1 min to 47.0, 48.2, 49.7, 51.3, 52.7, 54.3, 55.8, and 57.0 °C in an Eppendorf Mastercycler Nexus Gradient instrument. Each heat-treatment was preceded and followed by a 1 min incubation at 4 °C. Samples were mixed with 5 µl RNA sample buffer (New England Biolabs) and transferred into the sample wells. Electrophoresis was performed at room temperature and 5 V/cm for 3 h. Gels were then stained with Coomassie Brilliant Blue R-250 in 40 % ethanol and 10 % acetic acid for 10 minutes and destained in 40 % ethanol and 10 % acetic acid for 4 days. The gel was imaged using a Licor Odyssey (at 700 nm), and the resulting image was rendered and pseudo-colored using ImageJ software. The intensity of the bands of native virus, A and B particles was determined by densitometry. The obtained values were then normalized by setting the pixel value of the most intense band of the respective (sub)viral particle in the examined temperature interval to 100%. The value of a nearby, unstained region of the gel was assigned 0%.

**Negative stain electron microscopy**. Based on the results of above agarose gel electrophoresis, virus samples were prepared in an identical manner as described above to either preserve the native particles (47 °C), enrich for A particles (49.7 °C) or B particles (54.3 °C) followed by processing for negative stain electron microscopy. The procedure and identification of these (sub)viral particles based on the extent of capsid dye penetrability was done as specified in the Materials and Methods section of the main text.

**Supplementary Figure Captions**

**Supplementary Figure 1**

**Pentamers of RV-A2 subviral particles versus native virus reveal distinct changes in the microenvironment of TRP residues.** (a, b) Native RV-A2 (PDB: 1FPN), (c, d) A particle (PDB: 4L3B), and B particle (PDB: 3TN9) pentamers are displayed, viewed from above and below along the 5-fold symmetry axis. The inner and outer surfaces are color-coded according to the local hydrophobicity. Mapping was performed by using ChimeraX software, with blue indicating most hydrophilic, white equaling 0.0, and yellow being most hydrophobic (Kyte-Doolittle scale). TRPs are marked in red. TRP 2038 residues, which stack against the RNA in N and A particles, are marked with an asterisk. Boxes in panels b and d display a magnified view of the surface with two neighboring TRP 3027 residues. In both instances, the indole ring is shown as a stick model for better appreciation of their greater exposure to the solvent in (d) compared with (c). While not shown as inset, the same greater exposure of these TRPs is clearly evident in B particles (f).

**Supplementary Figure 2:**

**Fast heating shifts the (sub)viral particle transitions to lower temperatures.** (a) Native RV-A2 (N) was quickly heated (1 min ramp-up time) to temperatures covering the steep slope of the graphs in Figure 2a and the immediate pre- and post-transition regions. The population of (sub)viral particles (N, A, B) present at each selected temperature was then revealed by electrophoretic analysis. As found by us and others (Harutyunyan et al., 2013; Tsang et al., 2000), subviral A particle migrates faster than native particles despite their expanded capsid. Presumably, the highly porous protein shell is less efficient in shielding of the enclosed negatively charged nucleic acid compared to the almost insulating capsid of the native virions. In agreement, B particles lacking the polyanionic genome show only very low electrophoretic mobility. Taken from the observed broad band assigned to A particles, it is possible that in a fraction of these uncoating intermediates a variable part of the viral genome may have already partially exited the capsid as proposed for poliovirus (Levy et al., 2010; Tsang et al., 2000), with the different degrees of fully exposed negative charge leading to somewhat higher electrophoretic mobilities compared to “genuine” A particles. This particle assignment to the individual Coomassie blue-stained bands was independently confirmed by negative stain transmission electron microscopy (TEM) of samples heated to temperatures where each one of them dominated as outlined in the supplemental methods. (b) The presence of natural empty particles was evaluated by separating 10 µg of purified RV-A2 into a 15% sodium dodecyl sulfate-polyacrylamide gel; no significant amount of VP0, indicative of natural empties, was detected. Molecular weight marker and migration of structural proteins (VP1, VP2, and VP3) were depicted.

**Supplementary references**

Harutyunyan, S., Kumar, M., Sedivy, A., Subirats, X., Kowalski, H., Kohler, G., and Blaas, D. (2013). Viral uncoating is directional: exit of the genomic RNA in a common cold virus starts with the poly-(A) tail at the 3'-end. PLoS pathogens *9*, e1003270.

Levy, H.C., Bostina, M., Filman, D.J., and Hogle, J.M. (2010). Catching a virus in the act of RNA release: a novel poliovirus uncoating intermediate characterized by cryo-electron microscopy. Journal of virology *84*, 4426-4441.

Tsang, S.K., Danthi, P., Chow, M., and Hogle, J.M. (2000). Stabilization of poliovirus by capsid-binding antiviral drugs is due to entropic effects. Journal of molecular biology *296*, 335-340.
