# Supplementary figures and images for: nanoDSF: In vitro Label-Free Method to Monitor Picornavirus Uncoating and Test Compounds Affecting Particle Stability
Source: Front Microbiol. 2020 Jun 26;11:1442. doi: 10.3389/fmicb.2020.01442 (PMC7333345; doi:10.3389/fmicb.2020.01442)

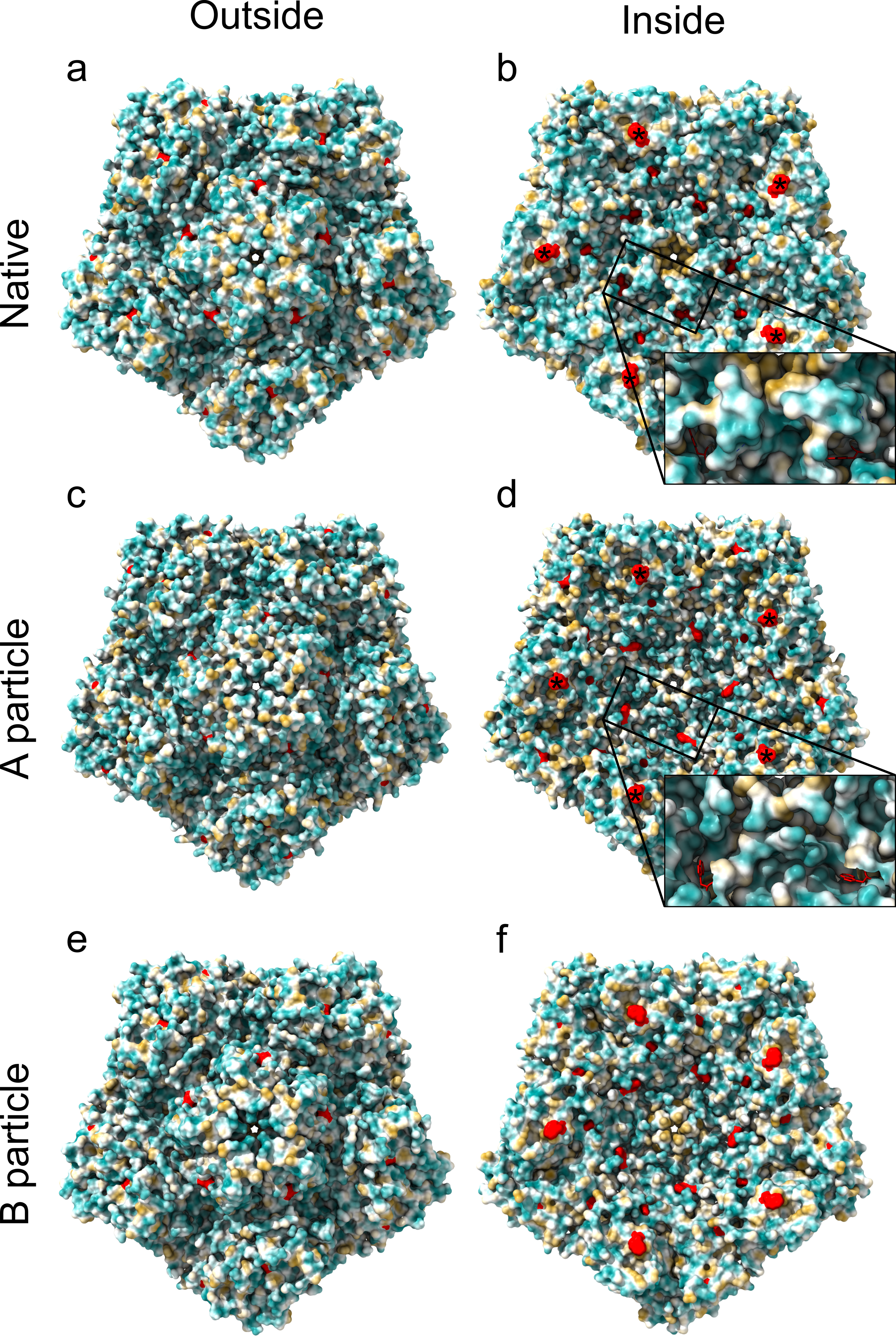

Supplement: Supplementary file 2 [file Image_1.TIF]

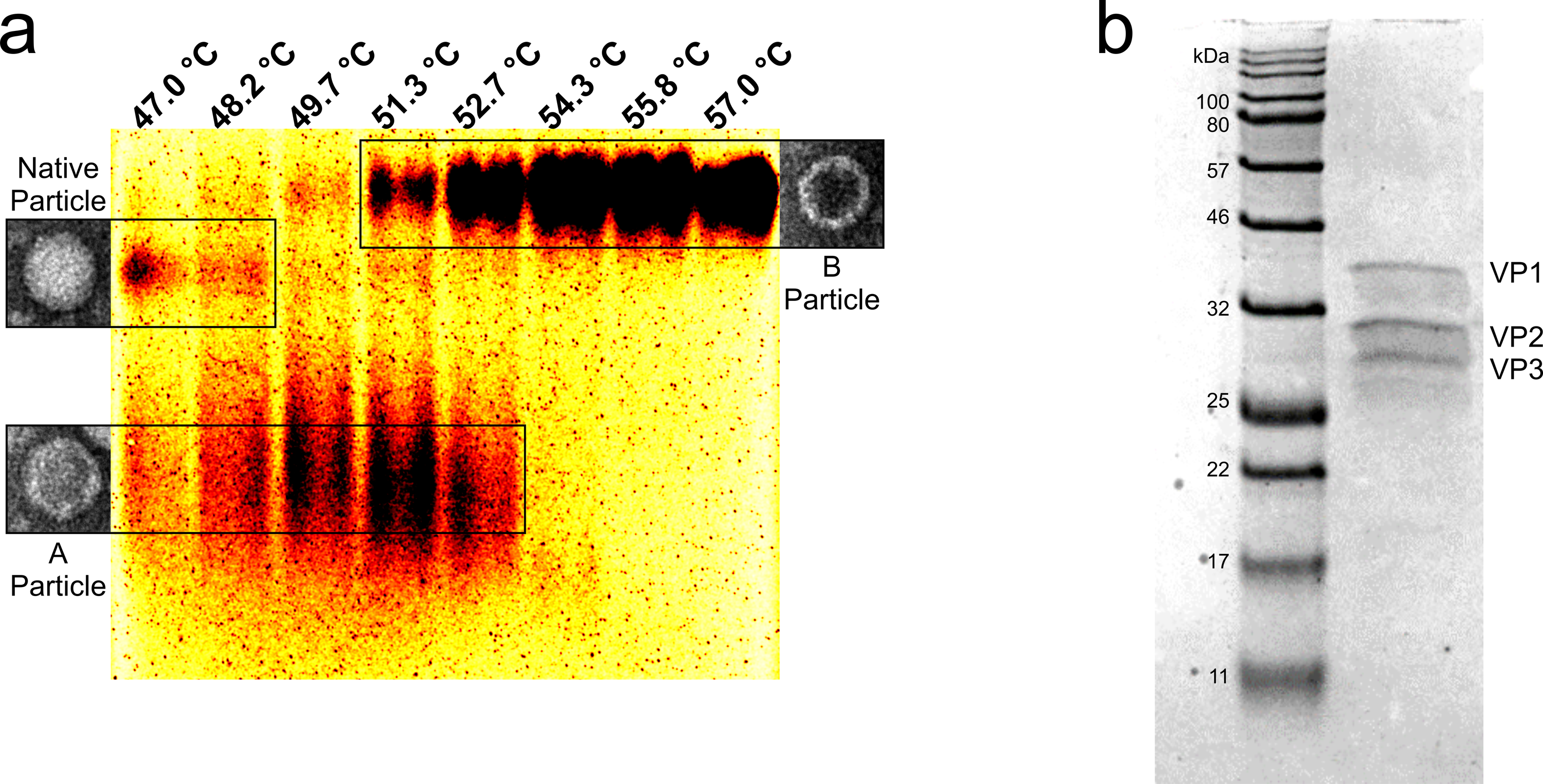

Supplement: Supplementary file 3 [file Image_2.TIF]
